# Regulation of Gene Expression Associated With the N6-Methyladenosine (m6A) Enzyme System and Its Significance in Cancer

**DOI:** 10.3389/fonc.2020.623634

**Published:** 2021-01-21

**Authors:** Shuoran Tian, Junzhong Lai, Tingting Yu, Qiumei Li, Qi Chen

**Affiliations:** ^1^ Fujian Key Laboratory of Innate Immune Biology, Biomedical Research Center of South China, College of Life Science, Fujian Normal University, Fuzhou, China; ^2^ The Cancer Center, Union Hospital, Fujian Medical University, Fuzhou, China

**Keywords:** N6-methyladenosine, m6A, m6A enzyme system, oncogene and tumor suppressor gene, cancer

## Abstract

N6-methyladenosine (m6A), an important RNA modification, is a reversible behavior catalyzed by methyltransferase complexes (m6A “writers”), demethylated transferases (m6A “erasers”), and binding proteins (m6A “readers”). It plays a vital regulatory role in biological functions, involving in a variety of physiological and pathological processes. The level of m6A will affect the RNA metabolism including the degradation of mRNA, and processing or translation of the modified RNA. Its abnormal changes will lead to disrupting the regulation of gene expression and promoting the occurrence of aberrant cell behavior. The abnormal expression of m6A enzyme system can be a crucial impact disturbing the abundance of m6A, thus affecting the expression of oncogenes or tumor suppressor genes in various types of cancer. In this review, we elucidate the special role of m6A “writers”, “erasers”, and “readers” in normal physiology, and how their altered expression affects the cell metabolism and promotes the occurrence of tumors. We also discuss the potential to target these enzymes for cancer diagnosis, prognosis, and the development of new therapies.

## Introduction

RNA methylation is one of the most important epigenetic modifications in RNA post-transcriptional modification. RNA methylation includes several types such as m6A, N1-methyladenosine (m1A), Eukaryotic 5-methylcytosine (m5C), 7-methylguanosine (m7G), RNA 2′-O-Methylation (Nm), etc. (1). m6A mainly occurs in the CDS region and the 3′-UTR region, especially in the region near the stop codon of mRNA. m1A and m5C modifications are initially found on tRNA and rRNA, but later, also on the mRNA. The m1A modification on mRNA mainly occurs near the initiation codon AUG, on the upstream of the first splicing site. It is found that m1A is often rich in GC bases but without strong sequence specificity (2). And m5C is mainly enriched in the untranslated region (5′-UTR and 3′-UTR), GC-rich region, and the vicinity of the AGO protein binding site, and it has the conserved sequence of AU(m5C)GANGU (3). In tRNA, m5C is mainly distributed in the variable arm and anti-cipher ring. In rRNA, m5C often appears in the region where associated with the translation activity (4). RNA methylation modification is a dynamic and reversible process regulated by RNA methylase and demethylase, and it needs the participation of RNA methylation binding protein. In the case of RNA m5C methylation, as a methyl donor, S-adenosine methionine (SAM) forms 5-methylcytosine (m5C) through the NSUN protein family (including NOL1, NOP2, and Sun) (5). The m5C methylated RNA exerts its biological function by binding to the reading protein ALYREF (6). TET3 may be the demethyltransferase of RNA m5C, and the specific mechanism of action needs to be further explored (7). Recent studies also found a synergistic effect between m5C and m6A in terms of the regulatory function of RNA. Nsun2-mediated m5C methylation and METTL3/METTL14-mediated m6A methylation can synergistically enhance the translation of P21 (8). Due to the space limitation, this review will focus on the recent progress on the m6A modification.

In 1974, m6A was first discovered in poly(A) RNA (9, 10), however, due to the scarcity of methodologies, research interest in m6A largely faded in the late 1970s (11). Since 2012, with the development of the antibody-based immunoprecipitation and high-throughput sequencing techniques for transcriptome-wide analysis of m6A, over 10,000 m6A peaks have been identified and validated in approximately 25% of human transcripts (12, 13). Modification of m6A has been found occurring in mRNA, non-coding RNA, 18S rRNA, 28S rRNA, U2-, U4-, and U6-spliceosomal RNA (14–17). In addition, following the development of a variety of antibody-independent detection methods and the sequencing depth reaching to a single base level, m6A modifications can be tested with high resolution in a variety of cellular environments (18, 19).

m6A is a methylation modification for the N6 position of adenylate, which is mainly found in the stop codon, 3′-UTR and long exon region (CDS). The modification site is often on the conserved sequence of RRACH (R = A or G; H = A, C, or U) (20–22). They are also found in many internal exon sequences and in 5′-UTR, which has been proven to participate in translation regulation. Typically, translation begins with the recruitment of the 43S ribosomal complex to the 5′ cap of mRNAs, however it seems that mRNAs containing m6A in their 5′-UTR can directly binds eukaryotic initiation factor 3 (eIF3) to initiate translation in the absence of the cap-binding factor eIF4e (23). Recent studies have shown that m6A modification of the first nucleotide near the cap of 7-methylguanine protects the mRNA from deacetylation and is involved in maintaining mRNA stability (24).

**Figure 1:**
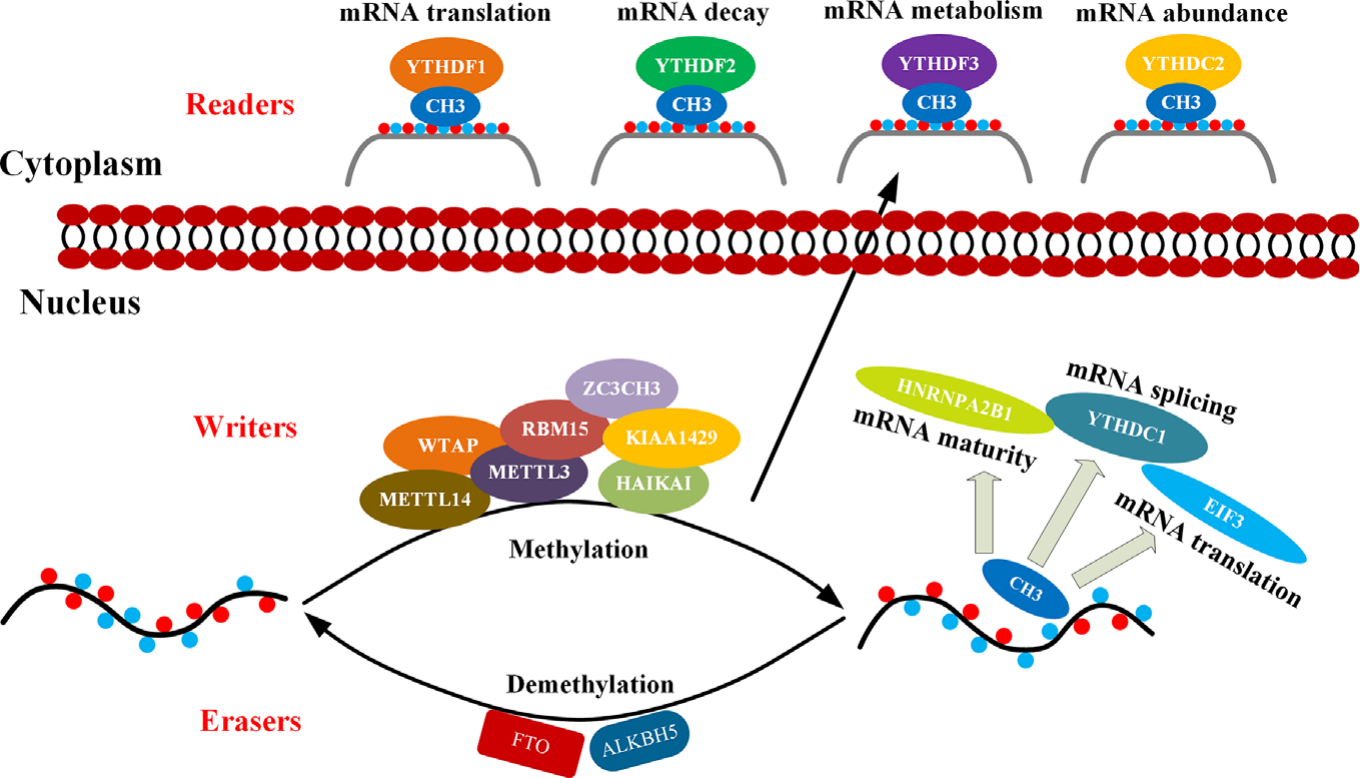
Mechanism of m6A. m6A methylation occurs through methyltransferase complexes: mainly by METTL3, METTL14 and WTAP complex (cofactors: KIAA1429, RBM15, ZC3CH3, and HAIKAI). The m6A modification is removed by demethylase FTO or ALKBH5. Reader proteins recognize m6A and determine target RNA fate (42).

The m6A modification process is dynamically reversible and regulated by the methylase complex, demethylase, and related binding proteins. The methyltransferase complex is mainly composed of methyltransferase like protein 3 (METTL3) (25), methyltransferase like protein 14 (METTL14) (26), Wilm’s tumor-associated protein 1 (WTAP) (27), the human homologous gene of Drosophila VIR protein (KIAA1429) (28), RNA binding motif protein 15 (RBM15) (29), and zinc finger CCCH domain protein 13 (ZC3H13) (30), as well as ZFP217 (31) and HAKAI (32). Most of them are able to catalyze m6A methylation modification *in vivo* and *in vitro*. METTL3 and METTL14 form the center of the complex. METTL14 has no catalytic domain but provides a backbone for RNA binding and assist METTL3 in increasing its catalytic efficiency. The important function of WTAP is to recruit METTL3 and METTL14 (33, 34). Therefore, these enzymes are collectively referred to as m6A “writers”. On the other hand, the obesity-related gene FTO and the demethylase ALKBH5 have been described as m6A “erasers” which are able to remove m6A modifications from RNAs (35, 36). In addition, the m6A “reader” proteins recognize methyl groups and promote downstream mRNA effects. The related proteins belong to YTH protein family, which include YTHDC1 found in the nucleus or YTHDC2 and YTHDF1/2/3 in the cytoplasm (37–41). Regulation and mechanism of the N6-methyladenosine (m6A) enzyme system can be seen in **Figure 1**. This review summarizes the latest progress in epigenetics research related to m6A methylation modification and its regulation involved in the occurrence and progression of multitudinous cancers.

## Structure and Function of m6A Enzyme System

### RNA Methyltransferase

#### m6A Methyltransferase Complex

METTL3 was first m6A modification enzyme identified, but METTL3 alone cannot perform catalytic enzyme activity (25). Later, WTAP and METTL14 were discovered by using tandem affinity precipitation combined with mass spectrometry (26, 27). Both METTL3 and METTL14 belong to a conserved family of methyltransferases which contain a MT-A70 domain (also known as MTD) that catalyzes the transfer of methyl group to adenosine (26). In the methyltransferase complex, METTL3 binds to methyl donor S-adenosine methionine (SAM) and catalyzes methyl transfer. Although not involved in catalytic reactions, METTL14 helps stabilize the structure of the catalytic center and acts as a scaffold for RNA binding (43). WTAP is functionally related to alternative splicing (44) and transports METTL3-METTL14 to the nucleus (27). In addition, KIAA1429 was also found to be a protein that participates in alternative splicing, interact with WTAP, and significantly affect the m6A methylation levels (28, 45). Besides, RBM15 has been found to interact with RNA substrates near the m6A site and introduces METTL3-METTL14 into RNA for the modification (29).

#### Other Components

More recently, Methyltransferase like protein 5 (METTL5) and Methyltransferase like protein 16 (METTL16) have also been shown to be m6A RNA methyltransferases. They both can catalyze m6A on certain structured RNAs (46, 47). METTL16 encodes SAM synthetase and can catalyze the modification of N6-methyladenosine at the 43rd position of U6 snRNA (47, 48). METTL16 can directly bind the hairpin structure in the 3′-UTR, and adjust the residence time and methylation efficiency immediately according to the changes in the intracellular SAM concentration, thereby completing the splicing of MAT2A mRNA and maintaining its stability (47, 49). Unlike METTL3-METTL14 heterodimers, METTL16-dependent m6A markers do not generally appear in the RRACH sequence motif but often exist at intron or intron-exon boundaries. Thus, METTL16 can take advantage of the sequence and structure specificity to bind corresponding RNA subunits (47, 48). Interestingly, one study suggests that METTL16 may play a multiplicity role in mRNA pre-splicing, acting as both m6A “writer” and m6A “reader”. As the “writer” of m6A, METTL16 rapidly methylated MAT2A mRNA in the presence of SAM, leading to intron retention and subsequent nuclear degradation. When SAM levels were low, METTL16 could remain on MAT2A mRNA for a long time, thus function as “reader” to enhance the preservation of intron splicing (48).

Recent studies have revealed that METTL5 is the m6A methyltransferase for 18S rRNA (46). Particularly, METTL5 forms a heterodimer with TRMT112 and provides the catalytic subunit to specifically methylate the 6th position of adenine in position 1832 of 18S rRNA (46). METTL5 may be essential for efficient translation, with profound implications for cellular function and pluripotency. Biallele variation of METTL5 may lead to lack of m6A modification on 18S rRNA, which has been shown to be associated with autosomal recessive intelligence and microcephaly in humans (50). These studies highlight the important role of METTL5 and ribosomal RNA modifications in gene expression, brain development and neurological function (51). ZCCHC4 is located at the nucleolus, where the ribosomes are assembled. ZCCHC4 has a potential m6A methyltransferase domain, with conserved catalytic motif “DPPF” and CCHC-ZNF domain, and can induce m6A methylation at 4220 in human 28srRNA (52, 53). When deleted, the accumulated level of m6A is significantly reduced in total rRNA, leading to unsteadiness in ribosome activity, which is associated with codon specific translation defects (54).

### RNA Demethylase

FTO and ALKBH5 are two currently known m6A demethylases and belong to the AlkB family of Fe (II)/α-ketoglutarate (α-KG) dependent dioxygenases. The AlkB demethylase family, which depends on a-KG, mainly catalyzes the oxidative demethylation of nucleic acid bases, and contains 9 members in mammals, including ALKBH1-8 and FTO (ALKBH9). Although these members have similar catalytic cores, they exhibit different substrate preferences (55). Both FTO and ALKBH5 specifically demethylate m6A on single-stranded RNA, but FTO demethylates m6A through three rounds of oxidation to produce two intermediate products hm6A and fm6A, while ALKBH5 directly converts m6A to adenosine with no intermediate products observed (56).

#### m6A Demethylase: FTO

In the early research, it was found that the m6A level in the mRNA of the FTO gene knockdown cells increased, and the opposite result occurs when FTO is overexpressed, suggesting the demethylation function of FTO (36). Later on, the existence of other substrates for FTO, such as N6, 2′-O-dimethyladenosine(m6Am), has been demonstrated (24). Therefore, FTO can mediate the demethylation of m6A and m6Am on RNA with polyA tail. The preference of FTO to m6A or m6Am is affected by its position in the cell. FTO has different positions in different cells. FTO in the nucleus can mediate the demethylation of m6A, while FTO in the cytoplasm can mediate the demethylation of both m6Am and m6A. In addition, FTO can also bind tRNA, function as a m1A demethylase of tRNA, and further affect the rate of protein translation (57).

#### m6A Demethylase: ALKBH5

ALKBH5, as a homologous protein of FTO in the ALKB family, is the second m6A demethylase identified. ALKBH5 knockdown results in a significant increase in the m6A level of mRNA, while ALKBH5 overexpression has an opposite effect (35). Different than FTO, ALKBH5 has no activity toward m6Am and appears specific to m6A (24).

### RNA Methylation-Modified Binding Protein

#### m6A-Binding Proteins: YTHDF1, YTHDF2, and YTHDF3

Members of the YTH family are highly conserved and contain an aromatic pocket of the YTH domain used to identify m6A-modified adenosine. These proteins are widely found in mammals, fruit flies, yeasts, and arabidopsis include YTHDF1/2/3 and YTHDC1/2 (38, 40, 41).

YTHDF1 was initially found to bind to the termination codon of m6A-modified transcripts, and its overall distribution is highly similar to that of m6A-modified transcripts (38). In addition, YTHDF1 can interact directly with the translation initiation complex, thus promoting the translation efficiency of m6A-modified RNA substrates. YTHDF1 is normally localized in the cytoplasm, but may also play a role in facilitating translation initiation and protein synthesis similar to that of eIF3 in the nucleus (58).

In 2014, YTHDF2 was first reported to act as a m6A binding protein to mediate m6A-modified mRNA degradation. Normally, YTHDF2 can co-localize with the adenylate enzyme complex and recruit CCR4-NOT, and the transcript of its target gene can be brought to the mRNA decay site (such as P body) through m6A modification, which then triggers the transcript adenylylation and degradation. YTHDF2 can alter its own cellular localization during heat shock stress (59).

Through tandem affinity precipitation combined with mass spectrometry and GST-pull down experiments, YTHDF3 and YTHDF1 were identified to interact with ribosomal 40S small subunit and 60S large subunit proteins (37). YTHDF3 and YTHDF1 have similar binding motif, mainly located at 3′-UTR. YTHDF3 together with YTHDF1, are cable to enhance the translation efficiency of target genes, showing a synergistic effect. In addition, YTHDF3 can mediate mRNA degradation through direct interaction with YTHDF2 (60).

#### m6A binding Proteins: YTHDC1 and YTHDC2

To investigate how m6A modification in the nucleus regulates selective splicing of mRNA, the researchers identified the m6A-binding protein YTHDC1, which is localized in the nucleus. YTHDC1 is responsible for splicing and output of mRNAs with m6A modification (61). YTHDC1 regulates selective splicing of RNA by interacting with SRSF3 (serine and arginine rich splicing factor 3) and SRSF10 (serine and arginine rich splicing factor 10). Either SRSF3 or SRSF10 can interact directly and competitively with YTHDC1. After YTHDC1 knockdown, the binding ability of SRSF3 to RNA and the nuclear localization signal of the protein were significantly weakened, while SRSF10 showed an opposite trend (62). YTHDC1 also plays an important role in the pre-mRNA processing of oocyte nuclei by interacting with the pre-mRNA 3′-end processing factors CPSF6, SRSF3 and SRSF7 (40). As the largest member of the YTH protein family, YTHDC2 preferred to bind a specific motif (GGACU) with m6A modification, thus accelerating the mRNA degradation and enhancing the translation efficiency (41).

#### Other m6A Binding Proteins

Other m6A binding proteins, including IGF2BPs (the family of insulin-like growth factor mRNA binding proteins), were identified by RNA pull-down method. In contrast to the function of YTHDF2/3, IGF2BP1/2/3 protects m6A-modified mRNAs in P-body and stress particles from degradation, and can facilitate mRNA translation by interacting with embryonic lethal abnormal vision (ELAV) such as RNA-binding protein 1 (HuR), matrix protein 3 (MATR3) and poly(A) binding protein cytoplasm 1 (PABPC1) (63, 64).

In the recent studies, HNRNPA2B1 of the HNRNP (splicing factors heterogeneous ribonucleoprotein) protein family is also considered to be an m6A binding protein. But it is different from YTHDC1 in that it cannot directly bind to the m6A. It usually activates the downstream pathway of pri-miRNA or participates in the processing of pre-miRNA. HNRNPA2B1 can recognize the binding of m6A-modified RGAC (R = A or G) motif in the miRNA transcript subset, and further exert alternative splicing effects by recruiting microprocessor complexes to promote miRNA processing. The m6A modification on pre-mRNA can recruit HNRNPA2B1, or increases the accessibility of the lateral RNA sequence with HNRNPC and HNRNPG by changing the local structure, thus becoming a “m6A switch” (65).

In addition, METTL3 may also have a function independent of its methyltransferase activity and act as a reader in the cytoplasm. METTL3, located near the termination codon of the mRNA, can interact with the 5′ cap by binding to elF3h, which promotes the ring formation of the mRNA, thereby promoting mRNA translation (66).

## Roles of The m6A Enzyme System in Human Cancers

Although m6A modification does not alter the normal pairing and coding functions of nucleotide sequence, it can affect gene expression extensively by interacting with various m6A modulating proteins. These proteins are involved in almost all processes of m6A-induced RNA metabolism, including mRNA translation, degradation, splicing and play important roles in cell function, development, and cancer progression (67). In addition, m6A modification can also affect the cleavage, transport, stability, and degradation process of miRNA, LncRNA and circRNA. Recent studies have revealed that the overall level of modification of m6A and its associated expression levels of regulatory proteins as well as the expression of affected non-coding RNA are often dysregulated in many types of cancer and can play an important role in the occurrence, progression, metastasis, drug resistance, and recurrence of cancer (68).

### Interaction Between m6A Enzyme System and mRNA in cancer

#### METTL3

METTL3 can affect cancer progression both positively and negatively (**Table 1**). In lung adenosarcoma, METTL3 promotes tumor cell growth, survival, and proliferation by promoting the translation of EGFR, TAZ, MK2, DNMT3A, and BRD4 (69–71). In hepatocellular carcinoma, METTL3 affects the occurrence of cancer by targeting SOCS2, a tumor suppressor gene, and m6A degradation mediated by YTHDF2 (72). In acute myelogenous leukemia (AML), METTL3 controls myeloid differentiation of normal hematopoietic and leukemia cells by promoting m6A-mediated translation of apoptotic genes such as C-MYC, BCL-2, and PTEN (73).

**Table 1:** Functions of m6A writers in cancer.

m6A Enzyme system	Cancer type	Overexpressed or Underexpressed	Target(s)	Changes in the behavior of tumor cells	Reference
**Wrirers**					
METTL3	lung adenocarcinoma.	Overexpressed	EGFR, TAZ, MK2, DNMT3A and BRD4	Promote the growth, survival, and proliferation	(69–71)
	breast carcinoma	Overexpressed	The positive feedback loop HBXIP/LET7G/HBX2P	Promote cell proliferation	(71)
	hepatocellular carcinoma	Overexpressed	SOCS2	Affect the occurrence of cancer through YTHDF2-mediated degradation of m6A	(72)
	acute myelogenous leukemia	Overexpressed	C-MYC, BCL-2 and PTEN	Promote the survival and proliferation	(73)
	endometrial carcinoma	Underexpressed	AKT signaling	Promotes cell proliferation	(74)
	endometrial carcinoma	Underexpressed	ADAM19, EPHA3 and KLF4/CDKN2A, BRCA2 and TP53	Promote the growth and self-renewal	(75)
	renal carcinoma	Underexpressed	Cell cycle	Promote cell proliferation, migration, and invasion	(76)
	gastric carcinoma	Overexpressed	H3K27-METTL3-HDGF-GLUT4/ENO2	Glycolysis	(77)
	hepatocellular carcinoma	Underexpressed	mTOR	Glycolysis	(78)
	colorectal cancer	Overexpressed	HK2/SLC2A1	Glycolysis	(77)
METTL14	pancreatic carcinoma	Overexpressed	AMPK α, ERK1/2 and mTOR signaling pathways	Promote cell apoptosis	(79)
	acute myelogenous leukemia	Overexpressed	MYC and MYB	Promote cell proliferation	(80)
	breast carcinoma	Underexpressed		Promote tumor growth, angiogenesis, and tumor development	(81)
	endometrial carcinoma	Underexpressed	AKT signaling	Promote cell proliferation	(82)
	bladder carcinoma	Underexpressed	Notch1	Negative regulation in proliferation, metastasis, and tumor initiation	(83)
WTAP	malignant glioma	Overexpressed	EGF	Oncogene	(84, 85)
	acute myelogenous leukemia	Overexpressed	HSP90	Regulation in abnormal proliferation and arrested differentiation	(86)
	renal carcinoma	Overexpressed	CDK2	Promote cell proliferation	(87)
	hepatocellular carcinoma	Overexpressed	HUR-ETS1	Promote proliferation of hepatocellular carcinoma cells *in vitro* and *in vivo*	(88)
	High-grade serous ovarian cancer	Overexpressed	MAPK and AKT signaling pathways	Associated with proliferation of cancer cells and lymph node metastasis	(89)

In other types of cancer, such as endometrial cancer, METTL3 expression was found to be reduced, promoting proliferation by altering AKT signaling (74). In malignant gliomas. the deletion of METTL3 can lead to upregulation of oncogenes such as ADAM19, EPHA3 and KLF4, and downregulation of tumor suppressor genes such as CDKN2A, BRCA2 and TP53, thereby promoting the growth and self-renewal of gastric stump cancer. Deficiency of the METTL3 in renal carcinoma cell lines significantly promotes cell proliferation, migration and invasion, and induced cell cycle G0/G1 phase arrest (75). Therefore, METTL3 was identified as a suppressor or promoter in certain cancers and further studies have indicated that the m6A level was changing following the development of cancer, which may partly explain the contradiction (76).

When involved in the regulation of tumor behavior, m6A modification can not only affect cell proliferation and migration, but also, equally important, regulate intracellular energy metabolism, such as glycolysis (77). Some data show that METTL3 is related to abnormal glucose metabolism and mTOR signaling pathway thereby is directly involved in the regulation of glycolytic activity in hepatocellular carcinoma. The down-regulation of METTL3 can weaken the glycolysis ability and cooperate with the glycolysis inhibitor 2-deoxyglucose (2-DG) to inhibit tumor growth *in vitro* (78). In addition, METTL3 is also shown to induce colorectal cancer relying on glycolysis. METTL3 can directly interact with the 5′ or 3′-UTR region of HK2 and the 3′-UTR region of SLC2A1 (GLUT1), relying on IGF2BP2 or IGF2BP3 to further stabilize these two genes and activate the glycolysis pathway (77). In gastric cancer, it can be found that m6A level is at a high level for the reason that METTL3 is activated by p300-mediated H3K27, which then leads to the enhancement of the stability of hepatocellular growth factor (HDGF) mRNA by m6A modification identified by “reader” IGF2BP3. HDGF can promote tumor angiogenesis, and nuclear HDGF activates the expression of GLUT4 and ENO2 which are associated with proliferation and liver metastasis of gastric cancer cells (90). In addition, studies have found that the enhancement of the “Warburg effect” (including glycolytic and lactic acid fermentation activity) can become a new sign of malignant cancer cells. The latest research shows that it is expected to become a new type of cancer treatment that reverses the Warburg effect and targets lactic acid, the end product of glycolysis, to reactivate oxidative phosphorylation (91). Therefore, the research on the relationship between m6A and glycolysis is becoming progressively significant. The studies elucidated above suggest that METTL3 and its related pathways may become a reasonable therapeutic target for cancer patients with high glucose metabolism.

#### METTL14

METTL14 also shows both promoting and anti-cancer effects (**Table 1**). Down-regulation of METTL14 expression interferes with AMPKα, ERK1/2 and mTOR signaling pathways, promotes apoptosis and can increase the sensitivity of pancreatic cancer cells to cisplatin (79). METTL14 promotes proliferation of cancer cells by promoting the translation of oncogenes MYC and MYB through m6A modification in AML, and its absence promotes the myeloid differentiation (80).

On the other hand, METTL14 expression is downregulated in glioblastoma, breast carcinoma, endometrial cancer, and bladder cancer cells and tissues. METTL14 expression level is down-regulated in breast cancer, in turn to promote tumor growth, angiogenesis and tumor occurrence and development (81). In endometrial cancer, METTL14 is deviancy due to mutation, which promotes cell proliferation by altering AKT signaling (92). METTL14 gene knockout promotes the proliferation, metastasis, and tumor initiation capacity of bladder cancer cell lines (TICs), this phenomenon disappears once METTL14 is overexpressed. Specifically, the m6A modification regulated by METTL14 is involved in the stability of Notch1 mRNA, which is a key factor in maintaining proliferation and invasion (83).

#### WTAP

WTAP has shown anti-cancer effect and been suggested as a novel therapeutic target (**Table 1**). In malignant gliomas, WTAP can regulate migration and invasion through EGF signaling, and closely related to glioma severity and postoperative survival rate of glioma patients (84, 85). WTAP is a novel client protein of HSP90 and promotes proliferation and clonal inhibition of differentiation in AML (86). In renal carcinoma, WTAP promotes cell proliferation, cell migration *in vitro*, and tumorigenesis *in vivo* by enhancing CDK2 expression (87). It was also found that WTAP is highly expressed in liver cancer tissue, and the progress of hepatocellular carcinoma (HCC) is related to the phenotype caused by WTAP deficiency. WTAP through m6A methylation modification leads to the post-transcriptional inhibition of HuR-ETS1, inhibits the E21-mediated P21/P27-dependent regulation of G2/M phase progression of hepatoma cell cycle, and promotes the proliferation and tumor growth of hepatoma cells (88). High expression of WTAP is associated with the overall survival of high-grade serous ovarian cancer (HGSOC). In ovarian cancer cell lines, after WTAP is down-regulated, cell proliferation and migration are significantly reduced, while the apoptosis rate is increased, which may be related to MAPK and AKT signaling pathways (89).

#### FTO

FTO also presents both pro- and anti-cancer effects (**Table 2**). It interacts with MYC and bHLH transcription factors to promote the proliferation of pancreatic cancer cells (93). FTO promotes proliferation and colony formation of lung cancer cells by increasing the expression of USP7 (ubiquitin-specific protease 7) (94).

**Table 2:** Functions of m6A erasers in cancer.

m6A Enzyme system	Cancer type	Overexpressed or Underexpressed	Target(s)	Changes in the behavior of tumor cells	Reference
**Erasers**					
FTO	pancreatic carcinoma	Overexpressed	MYC and bHLH	Promote cell proliferation	(93)
	Non-Small Cell Lung Cancer	Overexpressed	USP7	Promote cell proliferation	(94)
	Cervical squamous carcinoma	Overexpressed	β-catenin and ERCC1	Regulate the chemo-radiotherapy resistance of cervical squamous cell carcinoma	(95)
	Cervical squamous carcinoma	Overexpressed	E2F1 and MYC	Promote proliferation and migration	(96)
	Cervical squamous carcinoma	Overexpressed	BNIP3	In collaboration with YTHDF2 and promote cell proliferation, colony formation and metastasis	(97)
	hepatoma	Overexpressed	PMK2	Regulate the cell cycle and ectopic expression is associated with poor prognosis	(98)
	acute myelogenous leukemia	Overexpressed	ASB2 and RARA	Promote survival, proliferation, and cloning	(99)
ALKBH5	glioblastoma	Overexpressed	FOXM1	Promote cell self-renewal and its occurrence in brain tumor	(100)
	breast carcinoma	Overexpressed	NANOG	Maintain the tumor microenvironment	(101)
	Epithelial ovarian carcinoma	Overexpressed	EGFR-PIK3CA-AKT-mTOR signaling pathway and BCL-2	Enhance the autophagy in SKOV3 cells	(102)
	gastric carcinoma	Overexpressed	NEA-T1 and E2H2	Promote the invasion and metastasis	(103)
	lung adenocarcinoma	Overexpressed	FOXM1	Promote tumor progression	(104)
	pancreatic cancer	Underexpressed	WIF-1	Inhibit tumorigenesis and make tumor cells more sensitive to chemotherapy	(105)
	pancreatic cancer	Underexpressed	ATM-CHk2-p53/CDC25C	Inhibit the growth of tumor cells	(106)

In lung squamous cell carcinoma, FTO enhances the expression of M2F1 by reducing the level of m6A in the M2F1 transcript and maintaining the stability of mRNA, thus inducing carcinogenic function (107). FTO reduces the level of β-catenin mRNA through m6A modification, thus increasing the activity of resecting and repairing cross complementary group 1 (ERCC1) and enhances the resistance of the body to chemotherapy in cervical squamous cell carcinoma (95). FTO can interact with E2F1 and MYC transcripts, thus significantly reducing the translation efficiency of E2F1 and MYC, and promoting the proliferation and migration of cervical cancer tumor cells (96). FTO-mediated demethylation of m6A in the 3′-UTR of BNIP3 mRNA, a pro-apoptotic factor, and induced its degradation through YTHDF2 independent mechanism to promote breast cancer cell proliferation, colony formation and metastasis (97). FTO can induce demethylation and translation of the PKM2 gene and promote the occurrence of hepatocellular carcinoma (98). In AML, FTO affects the stability of ASB2 and RARA by lowering m6A levels to prevent bone marrow differentiation and promote survival, proliferation, and cloning (99).

In contrast, the deletion of FTO significantly promotes the proliferation and migration of bladder cancer including 5637 and T24 cells. It was found that the cells can be protected from the cytotoxicity induced by cisplatin when FTO expression was inhibited (108).

#### ALKBH5

ALKBH5 promotes the self-renewal of cells and regulates the occurrence of brain tumors by regulating FOXM1 in glioblastoma stem cells (100). In ovarian cancer, ALKBH5 activates the EGFR-PIK3CA-AKT-mTOR signaling pathway, enhances the stability of BCL-2 mRNA, and promotes the interaction between BCL-2 and Beclin 1. Silencing ALKBH5 enhanced autophagy in ovarian cancer SKOV3 cells (102). ALKBH5, through m6A and NEA-T1, affects the expression of the polyclonal inhibitor complex subunit E2H2, thereby promoting the invasion and metastasis of gastric cancer cells (103). ALKBH5 regulates the mRNA level of FOXM1 by reducing m6A modification, participates in the generation of intermittent hypoxia (IH) tumor microenvironment, and promotes proliferation and invasion of lung adenocarcinoma cells (104).

On the other hand, the loss of ALKBH5 often indicates a poor prognosis for colon and pancreatic cancers. ALKBH5 can inhibit the invasion and metastasis of colorectal cancer cells (102). In pancreatic cancer, ALKBH5 relies on the demethylation of Wnt inhibitory factor 1 (WIF-1) and the activation of Wnt signal to inhibit tumorigenesis and make tumor cells more sensitive to chemotherapy (105). In another study of pancreatic cancer, ALKBH5 is shown to activate PER1 in an m6A-YTHDF2-dependent manner, and induce the activation of the ATM- CHk2-p53/CDC25C signaling pathway to inhibit the growth of tumor cells (106) (**Table 2**).

#### YTHDF1/2/3

The deletion of YTHDF1 can greatly reduce the translation efficiency of CDK2, CDK4 and cyclin D1, which are closely related to cell cycle regulation, and can effectively inhibit the proliferation of NSCLC cells and the formation of xenograft tumors. However, in clinical treatments, researchers have found that the absence of YTHDF1 often makes cancer cells resistant to cisplatin (DDP) (109). In another study, researchers also found that silencing YTHDF1 expression can significantly inhibit the Wnt/β-catenin pathway, thereby inhibiting the tumorigenicity of colorectal cancer cells (110). YTHDF1 binds with m6A-modified EIF3C (a subunit of protein translation initiation factor EIF3) mRNA to enhance EIF3C translation, and at the same time promotes the overall translation output, thereby promoting the ovarian cancer occurrence and transfer (58).

In prostate cancer, YTHDF2 can promote cancer cell migration. In pancreatic cancer, the expression level of YTHDF2 increases with the extension of the cancer stage, which can inhibit epithelial-mesenchymal transition by inhibiting YAP signaling (111). In lung cancer, YTHDF2 directly binds to the m6A modification site (3′-UTR) of glucose 6-phosphate dehydrogenase (6PGD) to promote the translation of 6-PGD and the proliferation of lung cancer cells (112). YTHDF2 is highly expressed in AML, down-regulates TNFR2 and protects AML cell apoptosis, which is crucial for the development of leukemia. Hematopoietic stem cells (HSC) have the ability to differentiate and self-renew, which involves the regeneration and degradation of a large amount of mRNA. In the cytoplasm, YTHDF2 can promote m6A-dependent mRNA decay under normal and stress conditions, and play an important role in HSC homeostasis and hematological stress (113). In testicular germ cell tumors, YTHDF3 maintains cancer cell phenotypes through m6A modification (114).

In hepatocellular carcinoma, YTHDF2 not only exhibits a cancer-promoting effect, but also can exert a cancer-suppressing effect. YTHDF2 promotes CSC liver phenotype and tumor metastasis by regulating the 5′-UTR m6A level of OCT4 mRNA (115). YTHDF2 directly binds to the m6A modification site of EGFR 3′-UTR, promotes the degradation of EGFR mRNA, and inhibits the proliferation of cancer cells and tumor growth (116). YTHDF2 silencing in human hepatoma cells or ablating in mouse hepatocytes can cause inflammation, vascular reconstruction, or cancer metastasis (117).

#### YTHDC2 and IGF2BPs

Other studies of YTH family expression disorders have emerged in recent years (**Table 3**). The expression level of YTHDC2 is positively correlated with the stage or metastasis of colon tumor by promoting translation of HIF-1α (118). In hepatocellular carcinoma, IGF2BP1 can bind and stabilize c-MYC and MKI67 mRNAs, increase the expression of c-MYC and MKI-67 proteins, which are effective regulators of cell proliferation and apoptosis, and thus participate in regulation of tumor progression (119).

**Table 3:** Functions of m6A readers in cancer.

m6A Enzyme system	Cancer type	Overexpressed or Underexpressed	Target(s)	Changes in the behavior of tumor cells	Reference
**Readers**					
YTHDF1	Non-Small Cell Lung Cancer	Overexpressed	CDK2, CDK4 and Cyclin D1	Inhibit cell proliferation and the formation of a xenograft tumor	(109)
	colorectal cancer	Overexpressed	Wnt/β-catenin	Inhibiting the tumorigenicity	(110)
	ovarian carcinoma	Overexpressed	EIF3C	Promote the occurrence and metastasis	(58)
YTHDF2	pancreatic carcinoma	Overexpressed	YAP signal transduction	Inhibition of epithelial mesenchymal transformation	(111)
	lung cancer	Overexpressed	Glucose-6-phosphate dehydrogenase	Promote cell proliferation	(112)
	hepatocellular carcinoma	Underexpressed	EGFR 3′-UTR	Inhibit the proliferation and growth	(116)
	hepatocellular carcinoma	Overexpressed	OCT4 5′-UTR	Promote CSC liver phenotype and tumor metastasis	(115)
YTHDF3	germinal cell tumor of testis	Overexpressed		Oncogene	(117)
YTHDC2	Colorectal carcinoma	Overexpressed	HIF-1α	Promote the metastasis	(118)
IGF2BP1	Hepatocellular Carcinoma	Overexpressed	C-MYC and MKI67	Regulate cell proliferation and apoptosis	(119)

### Interaction Between the m6A Enzyme System and Noncoding RNAs in Cancer

In addition to mRNAs, m6A has also been found in a variety of non-coding RNAs (ncRNAs), such as miRNAs, long non-coding RNAs (LncRNAs), circular RNAs (circRNAs), ribosomal RNAs (rRNAs), small nuclear RNAs (snRNAs)), and small nucleolar RNAs (snoRNAs), can have an important impact on their metabolism and function (120–123), suggesting the potential association between tumor and m6A-ncRNA modification (124). Here, we discuss the interaction between m6A modification and non-coding RNA by focusing on the functional relevance of m6A in cancer progression, metastasis, and drug resistance (**Table 4**).

**Table 4:** Functions of m6A regulators in ncRNA metabolism.

ncRNA	Molecule	Cancer type	Biological function	Effect	Reference
miR221/222	METTL3	Bladder Cancer	Promote pri-miRNA processing	Positive	(126)
miR-1246	METTL3	Colorectal Carcinoma	Promote pri-miRNA processing	Positive	(127)
miR-143-3p	METTL3	Non-Small Cell Lung Cancer	Promote pre-miRNA processing	Positive	(128)
miR-29a	WTAP	Glioblastoma	Regulatory gene expression	Negative	(129)
miR-34-5p/miR-181-5p	IGF2BP1	Ovarian, Liver and Lung cancer	Maintain SRF target genes expression	Negative	(130)
miR-491-5p	IGF2BP1	Non-Small Cell Lung Cancer	Regulatory gene expression	Negative	(131)
miR-29A-3P, miR-29B-3P, miR-222, miR-1266-5P, miR-1268a, and miR-671-3p	HNRNPA2B1	Breast Carcinoma	Alter miRNAs transcriptome	Bidirectional	(132)
Linc00958 and miR3619-5p	METTL3	Hepatic Carcinoma	Stabilize lncRNA and enable the ceRNA model	Positive	(134)
lncRNA-NEAT1	ALKBH5	Gastric Carcinoma	Promote interaction	Positive	(103)
LINC01234	HNRNPA2B1	Non-Small Cell Lung Cancer	Promote interaction	Positive	(135)
lncRNA-DANCR	IGF2BP2	Pancreatic Carcinoma	Stabilize lncRNA	Positive	(136)
XIST	WTAP/RBM15/RBM15B	Gene silencing on the X chromosome	Promote XIST -mediated gene repression	Positive	(29, 138)
XIST	METTL14/YTHDF2	Colorectal Carcinoma	Mediate XIST degradation	Positive	(139)
circNSun2	YTHDC1	Colorectal Carcinoma	Promote cytoplasmic export of circRNAs	Positive	(140)
circRNAs	YTHDF2	Innate Immunity	Mediate circRNA degradation	Negative	(141)

#### m6A methylation regulates miRNA processing

miRNAs are a type of non-coding single-stranded RNA composed of 21 to 25 nucleotides. They regulate the expression of related genes after transcription by forming RNA-induced silencing complex (RISC). RISC binds to the target mRNA 3′-UTR, inhibits post-transcriptional modification and translation or induces mRNA degradation (125).

In recent years, m6A has been observed in miRNAs targeting oncogenes or tumor suppressor genes and is involved in tumor progression by affecting the biogenesis or stability of miRNAs. Changes in METTL3 can affect the steady state level of various miRNAs such as miR-25, miR-93, miR126, miR-221/222, and miR-4485. METTL3 may have a carcinogenic effect in bladder cancer by interacting with the microprocessor protein DGCR8 and actively regulating the pri-miR221/222 process in an m6A-dependent manner (126). The upregulation of METTL3 is related to the abnormal changes of m6A and positively correlated with colorectal tumor metastasis. In this context, the METTL3/miR-1246/SPRED2 axis plays an important role and provides a new m6A  modification mode for the development of colorectal cancer (127). In another study, the cleavage of pre-miR-143-3p is m6A-dependent, and METTL3 can positively regulate the miR-143-3p/VASH1 axis, increase lung cancer angiogenesis, and thereby regulate the VEGFA degradation and depolymerization of tubulin, playing an important role in the progress of non-small cell lung cancer (NSCLC) (128).

Overexpression of miR-29a inhibits WTAP expression by down-regulating QKI6, inhibits PI3K/AKT and extracellular signal-related kinase pathways, thereby inhibiting cell proliferation, migration and invasion, but promotes apoptosis in glioblastoma (129). As a conservative oncogenic driver network, IGF2BP1 promotes SRF expression in a conservative, m6A-dependent manner by inhibiting miRNA-induced SRF mRNA decay, leading to enhanced transcriptional activity of SRF, promoting tumor cell growth and invasion. At the post-transcriptional level, IGF2BP1 regulates the expression of various SRF target genes (including PDLIM7 and FOXK1) (130). IGF2BP1 is considered to be the direct target of miR-491-5p, and its expression is significantly up-regulated in non-small cell lung cancer tissues and inversely correlated with miR-491-5p expression (131).

Interestingly, it has been found that the overexpression of HNRNPA2/B1 in breast cancer cells can have an impact on the miRNA transcription group. HNRNPA2B1 can promote the endocrine resistance of breast cancer cells by down-regulating mir-29a-3p, mir-29b-3p, and mir-222, up-regulating mir-1266-5p, mir-1268a, and mir-671-3p, to reducing the sensitivity to 4-hydroxytamoxiphenylamine and fulva statin (132).

#### m6A methylation regulates in long Noncoding RNA

In recent years, more and more evidence has shown that LncRNA can control gene expression and cell functions at the transcriptional and post-transcriptional levels (133).

In terms of regulating the structure and function of LncRNA, METTL3-mediated m6A modification can stabilize the transcription process and lead to upregulation of lnc00958 which can promote the expression of miR3619-5p and further upregulate the expression of HDGF, thereby promoting liver cancer fat generation and progress (134). ALKBH5 combines with lncRNA-NEAT1 to remove the m6A modification that occurs on it and then affects the expression of the polyclonal inhibition complex subunit, EZH2, which in turn affects the invasion and metastasis of gastric cancer cells (103). Compared with normal lung tissue, the expression of LINC01234 in NSCLC was significantly increased, and it was positively correlated with poor prognosis. Further studies have found that LINC01234 can interact with HNRNPA2B1, which in turn leads to the recruitment of DiGeorge syndrome critical region gene 8 (DGCR8), promoting cell proliferation *in vitro* and tumor growth *in vivo* (135). IGF2BP2 is highly expressed in pancreatic cancer patients with poor prognosis, and inhibition of IGF2BP2 can inhibit cancer cell proliferation. The experiments further demonstrate that IGF2BP2 can stabilize lncRNA-DANCR by enhancing m6A modification to stabilize DANCR RNA, thus promoting cancer cell proliferation (136).

LncRNA-XIST can mediate transcriptional silencing of genes on the X chromosome. XIST is highly methylated, and m6A modification is necessary for XIST-mediated gene silencing (137). The formation of m6A on XIST is mediated by RBM15 and RBM15B. These m6A methylation complexes are often recruited to the specific sites within XIST, resulting in the formation of m6A on the adjacent sites. In addition, the m6A reader YTHDC1 is also recruited to XIST to promote XIST-mediated gene suppression (29, 138). As mentioned above, the loss of METTL14 is related to the poor prognosis of colorectal cancer patients. XIST mRNA is recognized by the m6A reading protein YTHDF2 which are further brought to be methylated by METTL14 through m6A, leading to XIST degradation, and thereby inhibits the tumorigenesis and metastasis of colorectal cancer cells (139).

#### m6A Methylation Regulates in CircRNAs

CircRNAs are post-splicing products of the precursor mRNA and have extensive cell-type-specific m6A methylation characteristics (142).

The presence of m6A circRNA is corroborated by interaction between circRNA and YTHDF1/YTHDF2, the proteins that read m6A sites in mRNAs, and by reduced m6A levels upon depletion of METTL3. Despite sharing m6A readers and writers, m6A circRNAs are frequently derived from the exons that are not methylated in mRNAs, whereas mRNAs that are methylated on the same exons that compose m6A circRNA exhibit less stability in a process regulated by YTHDF2 (140). In terms of regulating cancer, YTHDC1 can participate in m6A modification of circNSun2 to increase its output to the cytoplasm. By forming the RNA-protein ternary complex of circNSun2/IGF2BP2/HMGA2 in the cytoplasm, circNSun2 enhances the stability of HMGA2 mRNA and promotes colorectal cancer cell infiltration and liver metastasis (141).

## Conclusions and Perspectives

m6A is key to regulating multiple biological processes by determining the fate of RNA. The m6A modification is precisely regulated by “writer”, “eraser” and “reader” as well as non-coding RNAs, and involves almost any step of mRNA metabolism, as well as ncRNAs processing and circRNAs translation. Although research on m6A is still in its infancy, more and more studies on m6A in cancer have shown that m6A modification and its related regulatory proteins play an important role in a variety of cancers (**Table 1**). Although there are some inconsistencies in the current literature, many of the proteins involved in m6A regulation have only recently been identified (including ZC3H13 and CBLL1) and have not been investigated in the context of cancer and require further detailed study. Interestingly, we might think that the methyltransferase and dimethyl transferase involved in m6A play opposite roles in a cancer. However, this is not always the case. The methylation enzyme complex composed of METTL3-METTL14-WTAP and the demethylase of FTO can often show the same effect in cancer (73, 76). Consistent with this, the DNA demethylase and DNA methyltransferase (such as TET2 and DNMT3A) are known to acting as antineoplastic factors in myeloid malignancies (143). In addition, they work synergistically to suppress the lineage differentiation of hematopoietic stem cells (144). Therefore, in the similar tumor microenvironment, methylases and demethylases may work coordinately by regulating different target genes to exhibit cancer-promoting or anti-tumor effects. When targeting the same gene, it may also trigger similar biological results through different regulatory pathways. In addition, the fate of RNA transcripts modified by m6A is usually determined by the reader protein. Different readers may target different transcriptomes. However, in some cases, they may preferentially bind to different regions of the same transcriptome, or even competitively bind to the same regions of the same transcriptome. Therefore, to better understand how m6A modification is involved in regulating mRNA, it is important to understand which regions of mRNA transcription products are modified by m6A and the type of readers that binds to the modified regions.

The important role of m6A regulatory proteins observed in a variety of cancers suggests that they may be potential therapeutic targets for cancer therapy. In fact, many studies have been focused on developing inhibitors of FTO due to it demethylates N6 -adenosine modified (m6A) sites and N6,2 -O-dimethyladenosine modified (m6Am) sites of mRNA, thereby influencing multiple mRNA related processes including transcript stability, alternative splicing, mRNA translocation, and protein translation (145–147). For the other m6A modulators, the situation can be more complicated. For instance, METTL3 may have diametrically opposite regulatory effects by affecting m6A modification in different cancers. METTL3 may also have other independent activities independent of its catalytic activity (70). Therefore, the development of inhibitors targeting the catalytic activity of METTL3 may not be sufficient to inhibit its overall function, and further research is required to carry out to unravel its precise function and regulation.

Clinically, maladjustment of m6A and its regulatory proteins is related to the diagnosis and prognosis of cancer. Abnormal expression of one or more m6A modified proteins may serve as a biomarker for diagnosis and prognosis. It is worth noting that m6A is involved in the regulation of immune responses (either innate immunity or adaptive immunity) (148), The combination of immune checkpoint inhibitors and m6A enzyme system inhibitors may provide new and more effective treatment strategies for cancer.

In summary, there are still many difficulties that need to be overcome in order to target the proteins involved in the m6A enzyme system and establish an effective treatment. Small-molecule inhibitors targeting multiple enzymes for cancer therapy, like other drugs, will require more *in vivo* trials to rule out harmful off-target effects in the hope of reducing toxicity and increasing therapeutic specificity. However, the emerging research on RNA modification has provided new insights into the development of cancer, as well as opportunities to develop new treatments.

## Author Contributions

ST drafted the manuscript. JL, QL, and TY collected and sorted out literatures. ST and QC revised the manuscript. All authors contributed to the article and approved the submitted version.

## Conflict of Interest

The authors declare that the research was conducted in the absence of any commercial or financial relationships that could be construed as a potential conflict of interest.
